# Variceal transection with endoscopic submucosal dissection as a rescue therapy for esophageal varices with severe scarring

**DOI:** 10.1055/a-2791-4765

**Published:** 2026-02-24

**Authors:** Yasunori Yamamoto, Hirohito Mori, Kazuki Niida, Masaaki Tange, Eiji Takeshita, Yoshiou Ikeda, Yoichi Hiasa

**Affiliations:** 1 Department of Gastroenterology and Metabology, Ehime University Graduate School of Medicine, Toon, Japan; 2 Department of Advanced and Innovative Endoscopy, Ehime University Graduate School of Medicine, Toon, Japan; 3 Department of Inflammatory Bowel Diseases and Therapeutics, Ehime University Graduate School of Medicine, Toon, Japan; 4 Endoscopy Center, Ehime University Hospital, Toon, Japan

Standard treatments for esophageal varices, such as endoscopic variceal ligation (EVL) and endoscopic injection sclerotherapy (EIS), can induce mucosal fibrosis and scarring 1 2 , which complicates retreatment at recurrence. Furthermore, refractory varices frequently reflect perforating veins not controlled by superficial ligation 3 . We present a case demonstrating the utility of variceal transection with endoscopic submucosal dissection (VTE 4 ) as a rescue therapy.

**Fig. 1:**
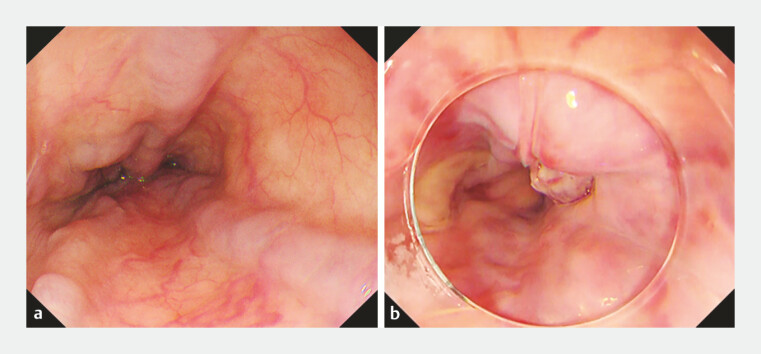
Pre-procedural endoscopic findings of the target esophageal varices. **a** Esophageal varices in the mid-esophagus. **b** Esophageal varices in the distal esophagus with post-endoscopic variceal ligation scarring.

**Fig. 2:**
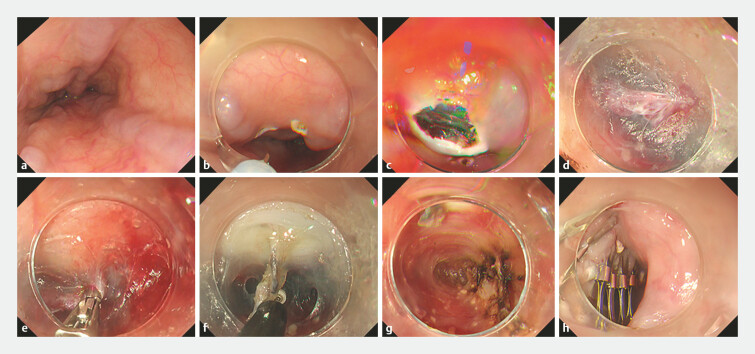
Key steps of the variceal transection with endoscopic submucosal dissection (VTE) procedure. **a** Identification of the target varices in the mid- to distal esophagus. **b** Marking of the mucosal entry site. **c** Creation of a submucosal tunnel parallel to the varices. **d** Endoscopic visualization of perforating veins within the submucosal tunnel. **e** Cauterization of the main perforating veins. **f** Transection and dissection of the varices from the underlying muscle layer. **g** Thorough cauterization of each perforating vein. **h** Final closure of the mucosal entry site with clips.

**Fig. 3:**
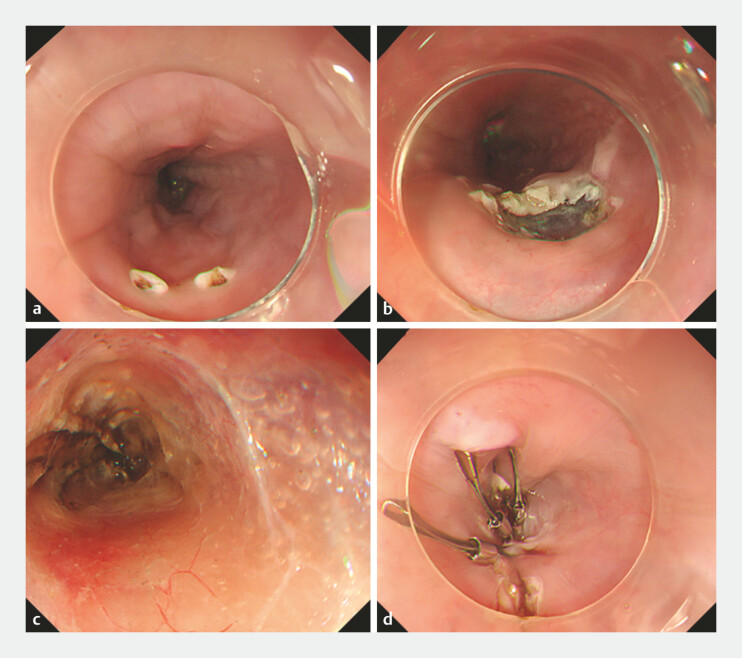
Application of the VTE procedure to a separate varix. **a** Marking the mucosal entry site near the target varix. **b** Creation of a submucosal tunnel. **c** Cauterization of perforating veins. **d** Closure of the entry site with clips. VTE, variceal transection with endoscopic submucosal dissection.

**Fig. 4:**
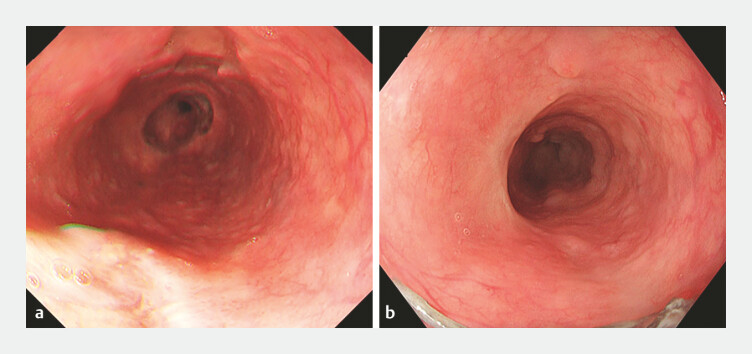
Post-procedural endoscopic findings showing the disappearance of esophageal varices. **a** Postoperative day 14. **b** Postoperative day 90.

A 71-year-old man with alcoholic liver cirrhosis had recurrent variceal ruptures despite prior EVL and EIS. After EVL achieved hemostasis for the latest rupture, extensive scarring made additional standard endoscopic therapy technically challenging ( Fig. 1 ). Retrograde obliteration was not feasible because no accessible shunts were identified, and a transjugular intrahepatic portosystemic shunt was unsuitable because of severe hepatic dysfunctions and insurance restrictions. Because the main obstacle was not controlling the portal pressure, but rather the technical inability to perform additional treatments on scarred mucosa, VTE was selected to dissect the scarred mucosa and coagulate feeding perforating veins ( Fig. 2 , Fig. 3 and Video 1 ). First, a small mucosal incision was created with a Dual Knife (KD-650Q; Olympus), avoiding the varix. Then, a submucosal tunnel was created parallel to the variceal trunk. The tunnel enabled the direct identification of feeding perforating veins. Each perforating vein was cauterized from the submucosa toward the outer wall using hemostatic forceps to interrupt blood flow. Smaller feeding vessels were sequentially coagulated under direct vision. Finally, the mucosal entry site was closed with clips. No delayed bleeding or perforation occurred. Endoscopic examinations on postoperative days 14 and 90 confirmed the sustained disappearance of the esophageal varices ( Fig. 4 ).

Variceal transection with endoscopic submucosal dissection (VTE): a successful rescue therapy for esophageal varices with severe scarring.Video 1

VTE may be a therapeutic option for esophageal varices when severe mucosal scarring prevents effective conventional treatment. By directly ablating feeding perforating veins in refractory cases, VTE addresses a key limitation of standard treatments.

Endoscopy_UCTN_Code_TTT_1AO_2AD
